# The COVID-19 laboratory response in Timor-Leste: a story of collaboration

**DOI:** 10.1016/j.lansea.2023.100150

**Published:** 2023-01-13

**Authors:** Nevio Sarmento, Endang Soares da Silva, Ismael Barreto, João C. Ximenes, Julia M. Angelina, Dircia M. Correia, Silvia M. Babo, Ari Jayanti P. Tilman, Antonio Salles de Sousa, Elisabeth Hornay, Lourenço C. Ico, Filipe de Neri Machado, Maria Varela Niha, Susan Ballard, Chantel Lin, Benjamin Howden, Rob Baird, Johanna Wapling, Lucsendar Alves, Tessa Oakley, Ian Marr, Anthony D.K. Draper, Paul Arkell, Heidi Smith-Vaughan, Nicholas S.S. Fancourt, Jennifer Yan, Joshua R. Francis

**Affiliations:** a Global and Tropical Health Division, Menzies School of Health Research, Charles Darwin University, Dili, Timor-Leste; b Molecular Diagnostic Laboratory, National Health Laboratory, Dili, Timor-Leste; c World Health Organization, Timor-Leste Country Office, Dili, Timor-Leste; d Departamento Vigilância e Epidemiologia, Ministério da Saúde, Dili, Timor-Leste; e Microbiological Diagnostic Unit Public Health Laboratory, The University of Melbourne at the Peter Doherty Institute of Infection and Immunity, Melbourne, Australia; f Territory Pathology, Royal Darwin Hospital, Darwin, Australia; g Northern Territory Centre for Disease Control, Darwin, Australia; h National Centre for Epidemiology and Population Health, Australian National University, Canberra, Australia; i Imperial College, London, United Kingdom

**Keywords:** Timor-Leste, COVID-19, SARS-CoV-2, Laboratory response, Pandemic, Low-resource, Collaboration

## Abstract

Timor-Leste is a small nation of 1.3 million people which shares a land border with Indonesia and is 550 km from Darwin, Australia. It is one of the poorest nations in Asia. The National Health Laboratory (NHL) and its network of smaller laboratories in Timor-Leste had limited capacity to perform molecular diagnostic testing before the coronavirus disease 2019 (COVID-19) pandemic began.

With the support of international development partners, the NHL rapidly expanded its molecular testing service. From March 2020 to February 2022, over 200,000 molecular tests were performed; COVID-19 testing sites were established in hospital and community health center laboratories and all 13 municipalities, and the number of scientists and technicians at the molecular diagnostic laboratory at the NHL increased from five to 28 between 2019 and 2022.

Molecular diagnostic testing for COVID-19 was successfully established at the NHL and in the municipalities. The molecular diagnostic laboratory at NHL is now equipped to respond to not only large-scale COVID-19 testing but also laboratory detection of other infectious diseases, preparing Timor-Leste for future outbreaks or pandemics.

## Introduction

Timor-Leste is a small nation of 1.3 million people that shares a land border with Indonesia and is 550 km from Darwin, Australia.^1^ Timor-Leste only recently achieved independence for the second time in 2002 and is one of the poorest nations in Asia.^2^

During the coronavirus disease 2019 (COVID-19) pandemic, the National Health Laboratory (NHL) of Timor-Leste needed to rapidly increase its capacity to perform molecular diagnostic testing. Globally, it was clear that molecular detection of severe acute respiratory syndrome coronavirus 2 (SARS-CoV-2) would be an essential component of national public health responses, including in Timor-Leste's nearest neighbours Indonesia,^3^^,^^4^ and Australia.^5^ Timor-Leste's ability to establish molecular testing capacity for SARS-CoV-2 in the NHL as well as in satellite laboratories needed to overcome challenges such as limited human resource capacity, limited existing diagnostic platforms (machines), and difficulty procuring laboratory reagents and consumables.

The Molecular Diagnostic Laboratory at NHL (NHL-MDL), Timor-Leste was established in 2011 as part of National Pandemic Influenza Preparedness (PIP) laboratory response.^6^^,^^7^ It predominantly processed nasopharyngeal samples for influenza-like illness (ILI) and severe acute respiratory illness (SARI) surveillance. The NHL-MDL was a small (4 m × 6 m) laboratory room, employing five scientists and technicians. To support the PIP program, the NHL-MDL utilised a single real-time polymerase chain reaction (RT-PCR) ABI 7500 (Applied Biosystems) with limited testing capacity for influenza A/B and typing only (maximum of 500 tests/month). This capacity was insufficient for nation-wide influenza monitoring.

GeneXpert (Cepheid, Sunnyvale, CA, USA) platforms have been available in Timor-Leste since 2012,^8^ and were primarily utilised for tuberculosis (TB) testing (MTB/RIF). The Xpert Xpress SARS-CoV-2 assay received emergency use authorisation (EUA) in March 2020 but was only available in Timor-Leste in late 2020 due to procurement challenges resulting from high global demand.

In February 2020, NHL collaborated with Menzies School of Health Research (Menzies) and the World Health Organization (WHO) Timor-Leste Country Office to assess the capacity of NHL-MDL to implement RT-PCR testing for SARS-CoV-2 in country. The assessment concluded with the following recommendations: (i) Identify lead scientists and recruit scientists and technicians to work in the NHL-MDL; (ii) Create task allocation in NHL to respond to the demand of laboratory testing; (iii) Procure reagents and consumables required for RT-PCR detection of SARS-CoV-2 using the molecular and diagnostic platforms available in the NHL-MDL; (iv) Establish standard operating procedures (SOPs) for molecular detection of SARS-CoV-2, infection prevention and control (IPC), and general laboratory guidelines for pandemic response; (v) Maximise the use of the small molecular diagnostic laboratory space; and (vi) Plan for expansion of the molecular diagnostic laboratory.

After the World Health Organization (WHO) declared a pandemic on March 2020^9^ the Office of the Prime Minister of Timor-Leste issued a decree (15/PM/III/2020)^10^ to officially launch the Integrated Center for Crisis Management (abbreviated as CIGC in Portuguese). The function of the CIGC was to coordinate the COVID-19 pandemic response task force in Timor-Leste, which included an Executive Committee and nine designated pillars of pandemic response. The NHL was tasked to lead pillar five – Laboratory Response.^10^

This mini-review describes the COVID-19 laboratory response in Timor-Leste, led by the NHL in collaboration with local and international partners. It will describe the implementation of PCR testing for SARS-CoV-2, human resource strengthening, data management processes, and the expansion of testing to municipalities.

## Approaches

**Fig. 1:**
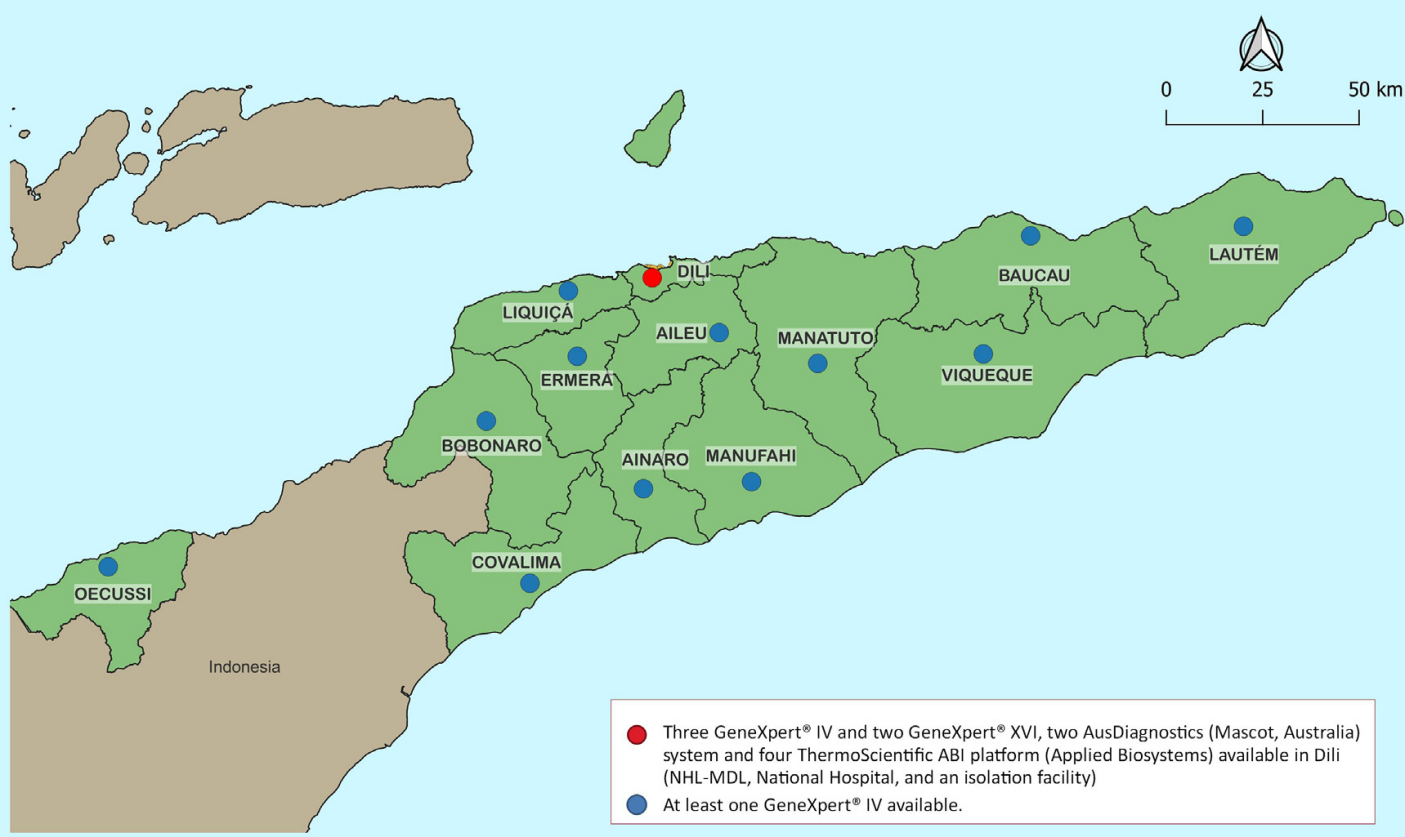
Type and location of molecular testing platforms in Timor-Leste, as of November 2022.

Accurate and widely available laboratory testing for SARS-CoV-2 was a crucial component of the response to the COVID-19 pandemic globally and in the region.^4^ Timor-Leste undertook a large, coordinated effort to establish SARS-CoV-2 testing, including test validation, expansion of molecular testing capabilities in Dili and the municipalities, increasing testing capacity, establishing a laboratory information management system (LIMS), training the molecular diagnostic workforce, developing a sample transportation model, and contributing to research and surveillance activities. This resulted in more than 200,000 SARS-CoV-2 tests, and 22,693 cases being detected as of February 2022 (Fig. 1). Increased testing capacity was instrumental in early detection and subsequent control of disease. Interventions such as mandatory isolation for all cases, quarantining for close contacts and international arrivals, and hospital admission and isolation for moderate and severe cases were implemented. General public health rules such as mandatory confinement (*confinamento obrigatório*) and sanitary fencing (*cerca sanitária*) to a particular municipality during periods of peak transmission were also implemented. The NHL and the PCR sentinel sites provided timely results to inform the public health decision making, with a 24-h specimen turn-around time.

### Establishment of testing capacity

In early 2020, procurement of molecular diagnostic items for SARS-CoV-2 testing was difficult, as border closures, disruption of supply lines and high global demand affected availability and access to reagents and consumables, none of which were produced in Timor-Leste. The government acted rapidly to overcome these obstacles, including Timor-Leste's limited access to global shipping lines. In February 2020, initial Real-time (RT) PCR reagents^11^ and SOPs for molecular detection of SARS-CoV-2 were supplied to NHL-MDL by the WHO.

At the NHL-MDL, Menzies worked alongside five Timorese scientists and technicians to perform testing of the initial samples collected from individuals with suspected COVID-19, collected from January to February 2020. Using the available QIAamp Viral RNA Mini Kit (Qiagen), PCR reagents and SOPs supplied by WHO, a testing method utilising the only available real-time PCR platform (ABI 7500, Applied Biosystems) was established in March 2020. The WHO and Menzies (with support from the Australian Government) purchased further molecular diagnostic systems and reagents to ensure ongoing testing capacity at NHL throughout the first six months of the pandemic. Increasing molecular diagnostic systems capacity was the only solution to diversify the supply chain and ensure continued testing capability in anticipation of a worsening of the pandemic.

Based on the initial assessment, Menzies School of Health Research employed a lead Timorese molecular scientist for the NHL-MDL as a Technical Advisor (TA) at NHL to lead the laboratory pandemic response. In collaboration with the Executives of NHL, the TA worked with Menzies and the WHO to establish three teams to operationalise the laboratory pandemic response: (i) sample collectors and transport teams; (ii) a labelling, sorting, and database management team; and (iii) a molecular testing and laboratory epidemiology dashboard team.

Anticipating the need for an increase in the laboratory's ability to process large number of samples, NHL recruited and trained 28 new scientists and technicians to join the COVID-19 laboratory response team. Due to different qualification levels, the 28 scientists and technicians were individually identified and appointed as scientists (8) and technicians (20). The Menzies TA provided training to the eight scientists on SARS-CoV-2 molecular detection methods, result interpretation, data analysis, updating daily laboratory epidemiology reports, and assisting in drafting SOPs. The 20 technicians who had limited knowledge of molecular diagnostic work were carefully trained, mainly performing extraction of nasopharyngeal swabs, mastermixing, and operating the molecular diagnostic systems. These 28 scientists and technicians played important roles for the expansion of tests in satellite laboratories, by training and mentoring their peers and daily support of testing, reporting and maintenance of equipment.

Prior to the pandemic, Menzies was collaborating with NHL on a project funded by the Fleming Fund Country Grant (UK Aid), which included plans to refurbish the existing NHL building to improve antimicrobial resistance (AMR) surveillance capacity and biosafety. Funds for laboratory refurbishment were then augmented by additional support from the Australian Government Department of Foreign Affairs and Trade (DFAT) to build a new physical containment level-2 (PC2) grade molecular laboratory for the COVID-19 response and future molecular diagnostics. The new PC2 molecular laboratory's approved dimension was 14 m × 8 m. Construction commenced in May 2020, and the building was inaugurated in December 2020. Through careful site management, the laboratory was able to function throughout this construction period, with no interruption to COVID-19 testing.

### Test validation

To ensure the quality of its molecular testing, the NHL collaborated with Territory Pathology, Royal Darwin Hospital, Darwin, Australia, and Menzies by sending batches of nasopharyngeal swabs from Timor-Leste to Australia for confirmatory testing. Overall, six batches (total of 240 samples) were sent from March to May 2020. The results demonstrated 100% concordance between testing in Timor-Leste and Australia. This quality assurance procedure ensured result integrity and allowed NHL to confidently process COVID-19 tests independently in Timor-Leste.

### Expansion of molecular testing in referral laboratories

With high demand for COVID-19 testing and anticipation of community transmission, the NHL-MDL expanded the molecular testing of COVID-19 to the municipalities outside the capital of Dili, utilising existing GeneXpert machines already used for MTB/RIF testing as well as installing new machines. To support NHL for expansion of COVID-19 testing, Menzies employed a municipality molecular TA for COVID-19 to oversee training, testing, result validation and reporting. Initially, the municipality TA travelled around the country to assess laboratory readiness. The district technical advisor then worked with the 28 scientists and technicians from NHL-MDL to provide bench-side training and mentoring to the technicians in the municipalities on the operation and maintenance of GeneXpert machines and the then active real-time data/result monitoring using GxAlert (SystemOne).

Before the pandemic, eight GeneXpert machines were available for TB testing (MTB/RIF) at NHL and several municipalities (Fig. 1; Table 1). The expansion of molecular diagnostics to the Timor-Leste municipalities started in June 2020 with the aim to establish testing capacity at *Hospital Nacional Guido Valadares* (HNGV), five regional referral hospitals (Baucau, Maliana, Maubisse, Oecusse and Suai), and eight Municipality Health Center Laboratories, including a COVID-19 isolation facility in Dili.

**Table 1:** Location and service of each molecular diagnostic platform in municipalities in Timor-Leste, November 2022.

Municipality (population)	Location of Analyser	Molecular testing capacity pre-COVID-19	Number of machine(s) pre-expansion	Number of machine(s) post-expansion	Funding source for expansion	Number of laboratory technician/scientist trained	Distance to Dili (Km)
Aileu (48,837)	Municipality Health Center	None	0	1	Ministry of Health Timor-Leste	2	44
Ainaro (63,136)	Referral Hospital	Yes, but limited to MTB/RIF	1	2	Ministry of Health Timor-Leste	6	110
Baucau (123,203)	Regional Hospital	Yes, but limited to MTB/RIF	1	2	Ministry of Health Timor-Leste	4	126
Bobonaro (97,762)	Referral Hospital	Yes, but limited to MTB/RIF	1	2	Korea International Cooperation Agency	4	150
Covalima (65,301)	Referral Hospital	Yes, but limited to MTB/RIF	1	2	Korea International Cooperation Agency	6	170
Dili (277,279)^b^	National Hospital and National Health Laboratory	Influenza A/B typing Xpert MTB/RIF, Xpert BCR-ABL for CML	1 ABI 7500 2 GeneXpert® IV and 1 GeneXpert® XVI	2 ABI 7000 and 2 ABI 7500 3 GeneXpert® IV and 2 GeneXpert® XVI	MoH Timor-Leste, DFAT-Menzies School of Health Research and WHO Timor-Leste	28	0
Ermera (125,702)	Municipality Health Center	None	0	2	Ministry of Health Timor-Leste	4	57
Lautem (65,240)	Municipality Health Center	None	0	1	Ministry of Health Timor-Leste	2	217
Liquiça (71,927)	Municipality Health Center	None	0	1	Ministry of Health Timor-Leste	2	30
Manatuto (46,619)	Municipality Health Center	None	0	1	Ministry of Health Timor-Leste	2	67
Manufahi a(53,691)	Municipality Health Center	None	0	1	Ministry of Health Timor-Leste	2	134
RAEOA/Oecusse (68,913)	Regional Hospital	Yes, but limited to MTB/RIF	1	2	Korea International Cooperation Agency	4	255
Viqueque (76,033)	Municipality Health Center	Yes, but limited to MTB/RIF	0	2	Ministry of Health Timor-Leste	4	184

a Number of population based on 2015 population census.

b Dili is the center of COVID-19 laboratory response, where 28 technicians/scientist were trained earlier anticipating for the increasing number of testing in the country.

As of November 2022, all municipalities have at least one working GeneXpert platform which can be used for COVID-19 and other viral respiratory testing, as well as increasing testing coverage for TB.

Initially, positive samples from municipalities were sent to LNS for confirmatory testing. However, ongoing communication between NHL and referral labs via WhatsApp (Meta Platforms, Inc.), including notification and discussion of results and training resulted in independent reporting in each facility. With discontinuation of GxAlert (SystemOne) NHL-MDL is currently working with Menzies School of Health Research to introduce Cepheid middleware c360, with a long-term goal to utilise the system to strengthen TB and HIV result notification from municipalities to Dili. The C360 will be interfaced with the NHL ***SchuyLab CGM*** (CompuGroup Medical, USA) for addition of GX results to patient's medical record.

In September 2020, the testing capacity of NHL-MDL was strengthened with the addition of an automated extraction platform (KingFisher Flex System, ThermoScientific). The NHL-MDL was further equipped with additional RT-PCR systems from Applied Biosystems and molecular diagnostic systems from AusDiagnostics (Mascot, Australia), and two GeneXpert® XVI (Cepheid, USA).

The estimated testing capacity for each machine varied depending on the type and number of the machine *in situ*, hours of operation, and available human resources. In Timor-Leste we estimate that if each machine operated for 10 working hours per day, a GeneXpert® IV could perform 53 tests each day; a GeneXpert® XVI could run 213 tests per day; an AusDx platform could run 240 tests per day; and a ThermoScientific ABI platform (Applied Biosystems) could run 940 tests per day. In the municipalities, methods such as sample pooling were performed^12^ in order to overcome limited human resources, conserve reagents and increase the number of individual samples processed. Sample pooling ceased in March 2021 once community transmission was widespread.

### Establishment of the laboratory information management system (LIMS)

Timor-Leste still lacks an electronic medical record system, and most hospital records remain paper based. For COVID-19, NHL-MDL initially utilised an online electronic spreadsheet (Google Sheets) for laboratory patient/client registry at NHL Timor-Leste. The spreadsheet document enabled simultaneous data entry and minimised errors associated with data entry and reporting. After implementation of the Fleming Fund Country Grant (UK Aid) in Timor-Leste, a license was purchased for the LIMS, ***SchuyLab CGM*** (CompuGroup Medical, USA). The LIMS was implemented in September 2020, initially in the microbiology department, however it was quickly utilised to develop a standardised report format for COVID-19 results that contained necessary information for surveillance and for international travel requirements, as per MoH guidelines. In 2022, the LIMS was implemented at Baucau and Suai (Covalima Municipality) referral hospitals for all other non-COVID-19 pathology tests and will eventually operate across all referral hospitals by the end of 2022.

The installation of a LIMS at NHL enabled streamlining of COVID-19 results, increased security and confidentiality, and facilitated collection of laboratory data epidemiological data analysis.

### Laboratory testing capacity

**Fig. 2:**
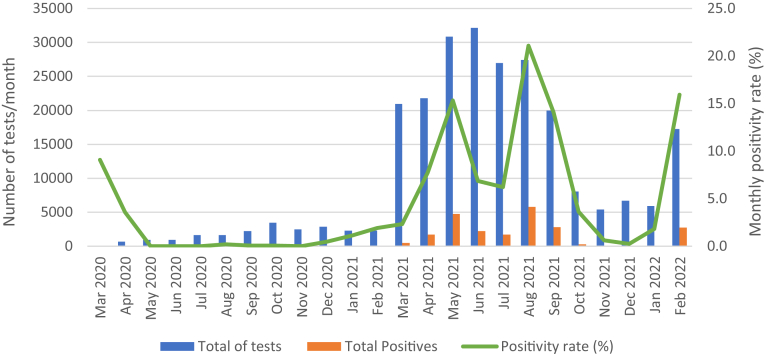
Monthly COVID-19 laboratory tests, total positives, and test positivity rates in Timor-Leste, March 2020–February 2022. Data sourced from National Health Laboratory and Surveillance Epidemiology Department, Ministry of Health Timor-Leste Databases (https://covid-19.ms.gov.tl/index2.php).

In the first half of 2020, NHL tested approximately 100 SARS-CoV-2 PCR tests per day. Testing numbers were limited using manual spin column-based nucleic acid extraction kits (QIAamp Viral RNA Mini Kit, Qiagen Diagnostics GmbH). With the addition of automated nucleic acid extraction systems and additional molecular diagnostics systems and reagents, testing capacity increased, peaking at 2500 tests per day (nationwide) in June 2021 (Fig. 2). As of February 2022, more than 200,000 molecular tests for SARS-COV-2 have been conducted at NHL-MDL.

From March 2020 to February 2021, Timor-Leste successfully prevented community transmission of COVID-19. This was largely due to swift government efforts in closing international borders, isolating cases in specific COVID-19 isolation facilities, and enforcing quarantine for all incoming travelers (in government managed quarantine facilities) and close contacts of cases (at home). The laboratory played an important role in testing and provision of quick turn-around-time results, assisting contact tracing and management of positive cases. The result of delaying community transmission for the first year of the pandemic, was that it afforded time for preparation for clinical and laboratory services. The first community transmission of COVID-19 cases was detected in March 2021 and as of February 2022, Timor-Leste had experienced three waves of COVID-19 community transmissions, firstly with the original variant (March 2021–June 2021), Delta variant (July to September 2021) and with ongoing Omicron variant transmission from January 2022.

### Laboratory sample transportation

At the beginning of the pandemic, all samples were transported to the Royal Darwin Hospital (RDH) laboratory in Darwin, Australia for testing, with a turn-around time of approximately seven days. As testing capacity was developed in Timor-Leste, the focus of laboratory sample transportation shifted to internal specimen transport, aimed at optimizing laboratory testing turn-around time.

The NHL-MDL is responsible for sample transportation in the capital Dili and five referral hospitals. In Dili, sample collectors worked at the main collection site (National Convention Center - NCC) external to the NHL, with a single sample transport car allocated. All five community health centers (CHC) in Dili, including isolation/quarantine facilities utilised ambulances to deliver samples to NHL. At the NCC between 500 and 1500 swabs were collected per day, stored in a cool box, and transported within 4–6 h of collection. All results in Dili were processed on the day of receipt with results typically available 24–48 h later. Before the expansion of RT-PCR to the municipal referral hospitals, ambulances transported samples to NHL and result turn-around time was typically 2–4 days. The delays from the municipalities are inevitable due to poor road conditions and the unavailability of vehicles. The expansion of the molecular services in June 2020 stopped the requirement of samples to be processed in Dili with each molecular site capable of testing more than 100 tests per day.

### Whole-genome sequencing (WGS)

Beginning in May 2020 the NHL sent COVID-19 positive samples for WGS to the Microbiological Diagnostic Unit Public Health Laboratory (MDU-PHL), at the University of Melbourne's Doherty Institute, in Melbourne, Australia. Convenience sampling of low cycle threshold (CT) positive samples was chosen to maximise sequencing yield. Until February 2022, nine batches with 804/1032 (78%) of samples were successfully sequenced. A detailed genomic sequencing report of SARS-CoV-2 positive samples from Timor-Leste will be published separately. The collaboration between the NHL-MDL and MDU-PHL identified circulating SARS-CoV-2 variants in Timor-Leste, including the variants of concern B.1.617.2 (Delta variant), first identified in July 2021, and the current (November 2022) Omicron BA.1 variant identified in January and February 2022. WGS contributed to identifying variants of concern and undertaking appropriate public health action in response.

### COVID-19 seroprevalence testing

The NHL-MDL, in collaboration with Menzies and Australia's National Center for Immunisation Research and Surveillance (NCIRS), conducted two separate seroprevalence studies: of COVID-19 in healthcare workers (HCWs) in Dili; and in residual serum samples nationwide. For HCWs there were two timepoints of testing between April–May 2021 and July–September 2021. The data from first testing timepoint showed 9.9% (32/324) of HCWs had anti-nucleocapsid (anti-N) IgG antibodies, due to previous infection. Subsequent testing during July–September 2021 showed 91.7% seropositivity, in which 86% were shown anti-spike IgG seropositivity. Acquisition of anti-N antibodies were also seen in 39.5% (30/76) of HCWs signalling an infection, post first dose of vaccine.^13^ The second study using residual blood was conducted in Dili and 4 regional sites of Timor-Leste between March and October 2021. During that period a total of 1652 residual serum samples were tested; prevalence of anti-spike IgG increased from 8.3% (95% CI 1.5–35.4%) to 87.0% (95% CI 77.7–92.8%) and anti-N IgG increased from 8.3% (95% CI 0.0–24.3%) to 45.5% (95% CI 34.8–56.3%).^14^ This study also identified an immunity gap in children and adults over 65 years; at the time children were not eligible for vaccination and adults over 65 years of age were under immunised despite being eligible. The results from these studies helped guide the COVID-19 vaccination program in Timor-Leste.

## Discussion

Establishment of molecular diagnostic service at NHL-MDL including expansion to the municipalities helped the low resource nation of Timor-Leste respond to the COVID-19 pandemic and has provided a legacy of ongoing molecular diagnostic capability in the country.

There were five main features of the COVID-19 laboratory response in Timor-Leste: political commitment from the government; collaboration or support from health development partners; improving procurement; increasing laboratory preparedness; and investing in human resources.

**Fig. 3:**
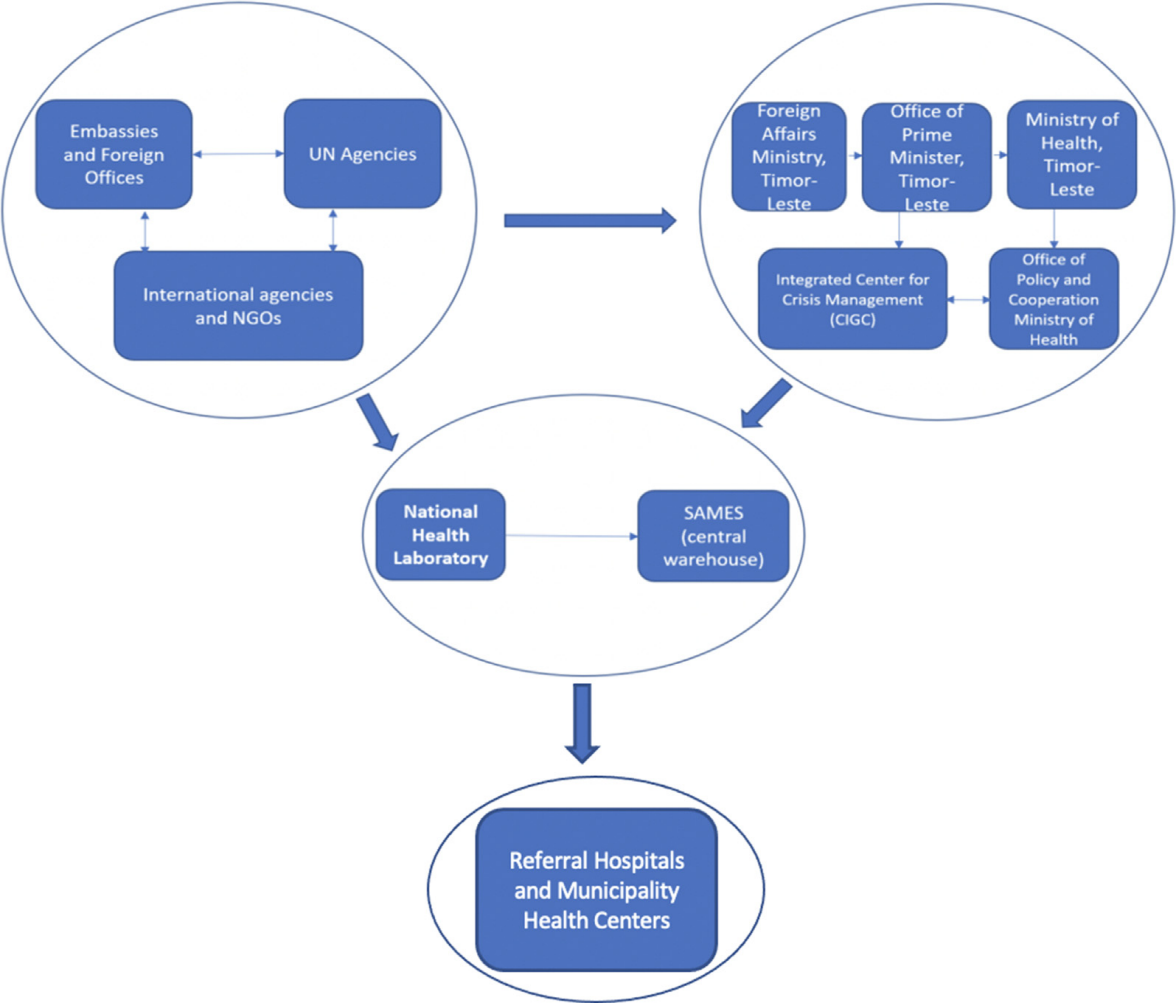
Schematic flow of external support and coordination work to assist the National Health Laboratory and municipality molecular diagnostic testing locations in Timor-Leste for COVID-19 laboratory (pandemic) response.

Global market pressures and limited freight options challenged the procurement of PCR reagents and consumables.^15^ Throughout the pandemic, laboratory inventory management was prioritised by the government, and the NHL and the central procurement agency worked together with partner organisations to ensure continuous supply and uninterrupted testing availability. Pragmatic approaches such as sample pooling enabled testing to continue even when faced with procurement challenges.^16^ Procurement was largely maintained due to efficient collaborations between the Timor-Leste Government, donors and partner organisations, including UN Organisations (WHO-TL,^17^ UNDP,^18^ UNICEF,^19^ and IOM^20^), and foreign embassies and associated organisations (DFAT,^21^ Menzies,^22^ USAID,^23^ Korea International Cooperation Agency (KOICA)^20^). This collaboration was coordinated by CIGC and the Office of Policy and Cooperation, Ministry of Health (Fig. 3). The NHL maintained control over decision-making regarding prioritisation of donated and purchased equipment and reagents, ensuring that efforts were not redirected to unwanted or unsuitable donations.

The NHL was appointed to lead pillar V (laboratory response) for COVID-19 in Timor-Leste through the CIGC. The NHL identified key weaknesses and sought collaboration with international counterparts to refurbish the laboratory, procure equipment and recruit human resources. Infrastructure investments in the newly built NHL-MDL provided additional space for more staff and new equipment, making it suitable for large-scale molecular testing in Timor-Leste. These structural improvements were coupled with investment in human resource training and capacity building, resulting in a cohort of 28 trained technicians and scientists capable of performing molecular testing across various platforms, techniques, and priority diseases.

The Fleming Fund Grant (UKAid) investment at the NHL by Menzies provided the foundation for more microbiological work, including capacity building, research and surveillance.^24^ The expansion of NHL-MDL directly benefits microbiology (AMR) work, where GeneXpert platforms are utilised for detecting resistant genes, including a recently detected *Acinetobacter baumannii* carrying New Delhi metallo-β-lactamase 1 (NDM-1) gene.^25^

Despite the incidence of COVID-19 decreasing to low levels, NHL-MDL has continued to monitor its spread and has integrated SARS-CoV-2 into the country's routine viral respiratory infection surveillance.

Challenges to COVID-19 laboratory response in Timor-Leste occurred due to the limited amount of time available to train a new workforce, but this was also overcome through collaboration between the MoH and international counterparts.

The NHL played an important role in controlling COVID-19 transmission in Timor-Leste. The strong collaborations between the Timor-Leste Government and international development partners have resulted in extended molecular testing capability which has the potential to include multiple respiratory pathogens, stool, blood, genital, and cerebrospinal fluid panels. Through smart investment, management, procurement and targeted funding channeling, the throughput has increased, and there are reasonable indications that the improvements will be sustained,^26^, ^27^, ^28^, ^29^, ^30^, ^31^ supporting high-quality molecular testing in Timor-Leste. There is ongoing collaborative work underway to establish dengue and malaria PCR testing capability to support outbreak control and elimination efforts, respectively.

## Conclusion

Timor-Leste has significantly increased its capacity to perform molecular testing for COVID-19 and is ideally positioned to capatilise and introduce testing for other organisms. The newly built NHL-MDL enabled large-scale testing capacity that supported the public health response during the pandemic and has facilitated the training and mentoring of an international standard molecular diagnostic service that can be sustained beyond the pandemic. The collaborative efforts between NHL and international partners were the key to these achievements and has established a sound model for future laboratory capacity building in Timor-Leste.

## Contributors

Nevio Sarmento (NS) drafted the manuscript. Endang Soares da Silva (ESDS) and Ismael Barreto (IB) provided technical, leadership and overall administrative support for COVID-19 laboratory response. João C. Ximenes (JCS), Julia M. Angelina (JMA), Dircia M. Correia (DMC), Silvia M. Babo (SMB), Ari Jayanti P. Tilman (AJPT), Antonio Salles de Sousa (ASDS), Elisabeth Hornay (EH), and Lourenço C. Ico (LCI) performed COVID-19 laboratory testing, training and assistance in expansion of COVID-19 laboratory testing to the municipalities. Felipe Neri de Machado (FNM), Maria Varela Niha (MVN), and Anthony D. K. Draper (ADKD) led the surveillance and epidemiology team for COVID-19 response in Timor-Leste. Susan Ballard (SB), Chantel Lin (CL), and Benjamin Howden (BH) provided molecular sequencing testing and interpretation, crucial for public health responses. Rob Baird (RB) supported the establishment of molecular testing capacity for COVID-19 in Timor-Leste through provision of quality assurance testing. Johanna Wapling (JW), Lucsendar Alves (LA), Tessa Oakley (TO), Ian Marr (IM), Paul Arkell (PA), Nicholas S S Fancourt (NSSF), Heidi Smith-Vaughan (HSV), Jennifer Yan (JY), and Joshua R. Francis (JRF) provided clinical, technical, procurement and overall guidance to NHL COVID-19 response. JRF is the senior author. All authors reviewed and approved the manuscript.

## Editor note

The Lancet Group takes a neutral position with respect to territorial claims in published maps and institutional affiliations.

## Declaration of interests

None declared.
